# Ivacaftor for the Treatment of Cystic Fibrosis Coexisting with Trisomy 21: A Case Report

**DOI:** 10.7759/cureus.6179

**Published:** 2019-11-18

**Authors:** Edward Charbek, Ghassan Kamel, Ravi P Nayak

**Affiliations:** 1 Internal Medicine, Saint Louis University School of Medicine, St. Louis, USA; 2 Internal Medicine: Critical Care, Saint Louis University School of Medicine, St. Louis, USA

**Keywords:** cystic fibrosis, ivacaftor, trisomy 21

## Abstract

The association between cystic fibrosis (CF) and trisomy 21, or Down Syndrome (DS) is rare, and it pertains a poor prognosis with the majority of patients dying in infancy. We report a case of a 28-year-old male with DS and moderate CF (ΔF508/G551D, FEV1 1.92 L, 60% predicted at the age of 18 years) diagnosed in childhood. The patient’s lung function continued to deteriorate over time (FEV1 nadir of 1.29 L), and he was started on ivacaftor in the year 2012 following ivacaftor release and approval. FEV1 and FVC improved significantly along with an increase in the patient’s body mass index. Ivacaftor potentiates the open-channel probability of the G551D-CFTR. It has been shown to improve lung function, symptoms, weight, and sweat chloride concentration and decrease the risk of pulmonary exacerbations in patients with severe pulmonary CF (G551D). Our case argues against the reported literature of poor prognosis when the two chronic diseases coexist as only one case report in the literature described a DS patient with CF surviving into adulthood. In our patient, treatment with ivacaftor resulted in an increase in FEV1 and weight that exceeded the response observed in the ivacaftor landmark trial. Genetic studies are underway to understand the genetic basis of the large variation in DS phenotypes, which is probably caused by allelic heterogeneity on multiple chromosomes. The latter may explain the enhanced response observed in our patient and suggests that although patients with concomitant DS and CF may have worse lung disease, their response to novel therapies may be intensified. Further studies are needed in this subset of patient population to better characterize CF with trisomy and other genetic disorders.

## Introduction

Cystic fibrosis (CF) is the most common genetic disorder in whites [1,2]. A mutation in the CF conductance transmembrane regulator (CFTR) gene, which codes for the CFTR protein, is responsible for the pathogenesis of the disease. There are more than 1,800 mutations identified in the pathogenesis of CF, which are classified into six classes. A class III mutation, G551D, is characterized by a dysfunctional CFTR protein on the cell surface that cannot transport chloride through its channel [3].

Down syndrome, caused by trisomy 21, is the most common chromosomal abnormality. It affects one in 600 to 800 live births. Respiratory conditions such as obstructive sleep apnea, asthma, pulmonary vascular disease, and parenchymal lung disease have been reported to be associated with Down syndrome [4,5]. The association between CF and Down syndrome is rare, and it pertains a poor prognosis with the majority of patients dying in infancy [6].

Ivacaftor, an oral CFTR potentiator, increases channel open probability (gating) of class III mutations. It has been shown to improve lung function, weight, and sweat chloride levels in clinical trials. Ivacaftor is an approved therapy for CF patients with several mutations such as G551D, G551S, G1349D, G970R, G178R, S549N, S1251N, and S1255P [7,8]. We report a case of a patient with Down syndrome and CF (ΔF508/G551D), who has successfully transitioned to the adult CF program at our institution and is being treated with ivacaftor.

## Case presentation

A 28-year-old male with Down syndrome that was diagnosed shortly after birth based on clinical characteristics. Frequent respiratory infections during infancy prompted further investigation that led to the diagnosis of CF based on a sweat test results of 74 mmol/L and genotype analysis of ΔF508/G551D. The patient had exocrine pancreatic insufficiency that affected his nutritional status during childhood as his weight was below 25th percentile. He was able to improve his weight and achieve adequate growth after introducing pancreatic replacement therapy. At the age of 18 years, he transitioned to the adult CF program at our institution, and he had moderate CF with FEV1 of 1.92 L, 60% predicted. The patient had no significant changes on chest radiography.

The patient had recurrent sinopulmonary infection and chronic *Pseudomonas aeruginosa* colonization; however, he did not have frequent CF-related pulmonary exacerbations requiring hospitalizations or home intravenous antibiotics.The patient’s lung function continued to deteriorate over time, and FEV1 reached a nadir of 1.29 L, 42% predicted. Subjectively, he was feeling more dyspneic and became limited in his daily activities. In May 2012, he started ivacaftor and followed regularly with spirometry measurements to monitor disease progression. His lung volumes, particularly FEV1 and FVC, improved significantly along with increase in his body mass index.

Table 1 demonstrates treatment response overtime. Improvement in respiratory symptoms was reported by the patient as well. In fact, he was able to conduct his daily activities with minimal limitations, and he started working part time at a fastfood restaurant. He did not require any hospitalization during the time he was on ivacaftor.

**Table 1:** Lung function and body mass index changes over time following treatment with ivacaftor FEV1: forced expiratory volume in one second, FVC: forced vital capacity, BMI: body mass index.

	FEV1 (L)	FEV1 %	FVC (L)	Weight (kg)	BMI (kg/m^2^)
Baseline	1.48	46	1.62	53.5	23.5
12 months	1.58	50	1.98	60.3	26.8
60 months	2.09	65	2.55	58.5	26.5

## Discussion

Ivacaftor potentiates the open-channel probability of the G551D-CFTR. It has been shown to improve lung function, symptoms, weight, and sweat chloride concentration and decrease the risk of pulmonary exacerbations in patients with severe pulmonary CF due to G551D mutation [8]. To our knowledge, this is the first case that demonstrates ivacaftor effectiveness in a patient with CF due to G551D mutation and Down syndrome.

The reported literature suggests poor prognosis when the two chronic diseases coexist as only one case report in the literature described a DS patient with CF surviving into adulthood [9-11]. In our patient, treatment with ivacaftor resulted in an increase in FEV1 and weight by 19 percentage points and 5 kg, respectively, which significantly exceeded the response observed in the ivacaftor landmark trial of 10.6 percentage points and 2.7 kg in FEV1 and weight, respectively [8]. 

Down syndrome is the most common recognizable genetic syndrome associated with immune defects [12] with an increase in susceptibility to recurrent respiratory infection due to abnormal components of the immune system [13,14]. Airway abnormalities such as laryngomalacia and tongue and tonsillar enlargement, the increased prevalence of gastroesophageal reflux disease, and congenital ear abnormalities are among the non-immunologic factors that increase the risk of respiratory infections in Down syndrome [15-18]. The increase risk of respiratory infections probably accounts for the magnified poor outcomes in CF patients with Down syndrome.

It is known for mutations on chromosome 21 to be more phenotypically apparent as in the early onset of Alzheimer's disease seen in Down syndrome patients. However, this association has not been studied in other chromosome mutations such as chromosome 7 in CF patients. Genetic studies are underway to understand the genetic basis of the large variation in Down syndrome phenotypes, which is probably caused by allelic heterogeneity on multiple chromosomes [19].

## Conclusions

CF and Down syndrome can occur concomitantly. Our patient had an enhanced response to ivacaftor which may suggest that although patients with concomitant Down syndrome and CF may have worse lung disease, their response to novel therapies may be intensified. Further studies are needed in this subset of patient population to better characterize CF with trisomy and other genetic disorders.
